# Endocardial Tip Cells in the Human Embryo – Facts and Hypotheses

**DOI:** 10.1371/journal.pone.0115853

**Published:** 2015-01-24

**Authors:** Mugurel C. Rusu, Cristian V. Poalelungi, Alexandra D. Vrapciu, Mihnea I. Nicolescu, Sorin Hostiuc, Laurentiu Mogoanta, Traian Taranu

**Affiliations:** 1 Division of Anatomy, Faculty of Dental Medicine, “Carol Davila” University of Medicine and Pharmacy, Bucharest, Romania; 2 MEDCENTER—Center of Excellence in Laboratory Medicine and Pathology, Bucharest, Romania; 3 Department of Obstetrics and Gynaecology “Dr.I.Cantacuzino” Hospital, “Carol Davila” University of Medicine and Pharmacy, Bucharest, Romania; 4 Division of Histology and Cytology, Faculty of Dental Medicine, “Carol Davila” University of Medicine and Pharmacy, Bucharest, Romania; 5 Laboratory of Molecular Medicine, “Victor Babeş” National Institute of Pathology, Bucharest, Romania; 6 Division of Legal Medicine and Bioethics, Department 2 Morphological Sciences, Faculty of Medicine, “Carol Davila” University of Medicine and Pharmacy, Bucharest, Romania; 7 Research Center for Microscopic Morphology and Immunology, Department of Morphology, University of Medicine and Pharmacy of Craiova, Craiova, Romania; 8 Division of Anatomy, Faculty of Medicine, “Gr.T.Popa” University of Medicine and Pharmacy, Iasi, Romania; Northwestern University, UNITED STATES

## Abstract

Experimental studies regarding coronary embryogenesis suggest that the endocardium is a source of endothelial cells for the myocardial networks. As this was not previously documented in human embryos, we aimed to study whether or not endothelial tip cells could be correlated with endocardial-dependent mechanisms of sprouting angiogenesis. Six human embryos (43–56 days) were obtained and processed in accordance with ethical regulations; immunohistochemistry was performed for CD105 (endoglin), CD31, CD34, α-smooth muscle actin, desmin and vimentin antibodies. Primitive main vessels were found deriving from both the *sinus venosus* and aorta, and were sought to be the primordia of the venous and arterial ends of cardiac microcirculation. Subepicardial vessels were found branching into the outer ventricular myocardium, with a pattern of recruiting α-SMA+/desmin+ vascular smooth muscle cells and pericytes. Endothelial sprouts were guided by CD31+/CD34+/CD105+/vimentin+ endothelial tip cells. Within the inner myocardium, we found endothelial networks rooted from endocardium, guided by filopodia-projecting CD31+/CD34+/CD105+/ vimentin+ endocardial tip cells. The myocardial microcirculatory bed in the atria was mostly originated from endocardium, as well. Nevertheless, endocardial tip cells were also found in cardiac cushions, but they were not related to cushion endothelial networks. A general anatomical pattern of cardiac microvascular embryogenesis was thus hypothesized; the arterial and venous ends being linked, respectively, to the aorta and *sinus venosus*. Further elongation of the vessels may be related to the epicardium and subepicardial stroma and the intramyocardial network, depending on either endothelial and endocardial filopodia-guided tip cells in ventricles, or mostly on endocardium, in atria.

## Introduction

The growth of the vascular system during development involves sprouting, migration and proliferation of endothelial cells (ECs) [1]. Sprouting angiogenesis is guided by a distinctive cell-type, the endothelial tip cell (ETC) [1–7] and is promoted by pro-angiogenic signals such as vascular endothelial growth factor (VEGF), that controls whether specific ECs become ETCs or trailing stalk cells [8, 9]. Neuropilin 1, which is a receptor for the VEGF-A, and is essential for normal angiogenesis, promotes ETC function [10]. During sprouting angiogenesis, the ETCs actin-rich filopodia penetrate the stromal compartment and are exposed to microenvironmental influences [2, 11, 12].

The development and patterning of the coronary vascular system is a relatively unexplored area with important consequences for human health [13]. Coronary vascular development occurs first by vasculogenesis and then subsequently by vascular sprouting. Hematopoietic commitment precedes formation of blood islands in the coronary vasculature [14]. It was considered that endocardial ECs form a large sheet without angiogenic sprouting into the myocardium [15]. However, Wu et al. (2012) recently established, by *in vitro* animal studies, that endocardial cells generate endothelia in coronary arteries. The endocardial cells are thus not terminally differentiated, but are angiogenic capable and able to form coronary endothelial networks [16]. The valuable contribution of these authors was analyzed and was considered that further work is needed in order to evaluate the contributions to the coronary tree brought by the endocardium, the proepicardium and the *sinus venosus* [17].

Few experimental studies studied the role of the endocardium in coronary vascular development [16, 18]. Endocardial angiogenesis was not assessed in humans. Moreover, the sprouting mechanism of endocardial angiogenesis was not evaluated *in situ*.

We aimed to evaluate whether endocardial cells of human embryos present an immune phenotype and a morphology specific to tip cells involved in endocardial sprouting. In addition, we explored the differences of the endothelial sprouts by using immunohistochemistry.

## Materials and Method

Six human embryos from legal abortions were collected immediately after the miscarriage. The lengths of these embryos varied between 12 and 29 mm, corresponding to 43–56 days embryos [19]. The study was conducted in accordance with the Oviedo Declaration (art. 18), regarding the research on embryos in vitro, and the relevant national Law (104/2003 regarding the manipulation of human cadavers and its metodological norms). The Ethics Committee of University of Medicine and Pharmacy of Craiova approved this study (Approval #54/20.03.2014). The participants provided their written informed consent to participate in this study. The samples were not procured from a tissue bank or donation centre.

Samples were formalin (10%)-fixed, sagitally oriented and paraffin-embedded. The samples were sectioned at 3 μm, and stained with Hematoxylin-Eosin to appreciate the general histology of tissues.

The following primary antibodies were used: **(1)** CD 34 (clone QBEnd 10, Dako, Glostrup Denmark, 1:50); **(2)** CD 105 (polyclonal, Thermo Scientific, Pierce Biotechnology, Rockford, USA, 1:50); **(3)** α-smooth muscle actin (α-SMA) (clone 1A4, Dako, Glostrup, Denmark, 1:50); **(4)** desmin (clone D33, Biocare Medical PM 036 AA, Biocare Medical, Concord, CA, USA, 1:100); **(5)** CD31 (clone JC70A, Dako, Glostrup Denmark, 1:50) and **(6)** vimentin (clone V9, Dako, Glostrup Denmark, 1:50).

Sections were deparaffinised, rehydrated and rinsed in PBS buffer solution at pH 7.4. Retrieval by incubation in specific buffer was completed as follows: (a) for CD34: EDTA, pH 9; (b) for the other antibodies: 0.01 M citrate retrieval solution, pH 6. The standard ABC technique used a DAB protocol. Appropriate blocking of endogenous peroxidase was completed before immune labelling (Peroxidazed 1, Biocare Medical, Concord, CA, USA). Sections incubated with non-immune serum served as negative controls (S1 Fig.). The immune labeled sections were counterstained with Hematoxylin.

The microscope slides were analyzed and micrographs were captured and scaled using a Zeiss working station: AxioImager M1 microscope with an AxioCam HRc camera and AxioVision digital image processing software (Carl Zeiss, Oberkochen, Germany).

## Results

**Figure 1:**
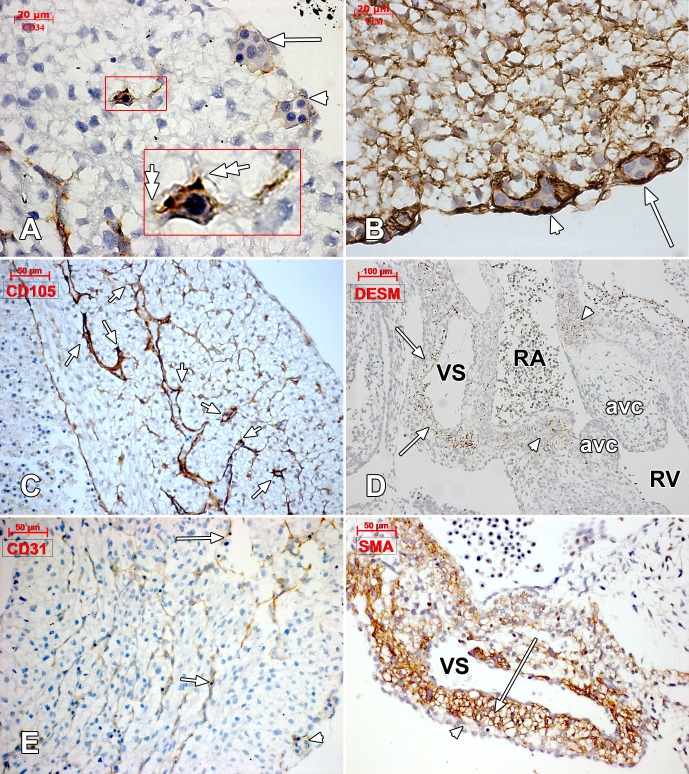
Human embryonic heart (43 days), CD 34, vimentin, CD105, desmin and CD31 immune labeling. Immune labeling with CD34 (A) and vimentin (B) antibodies of a 43 days human embryonic heart, oblique-sagittal cut at ventricle level. Corresponding epicardial vascular canals are indicated (white arrrows and white arrowheads). The walls of these canals seem to acquire a CD34-positive phenotype and are vimentin-positive. In (A) an active intramyocardial process of sprouting angiogenesis is detailed (inset), being guided by tip cells (double-headed arrows). CD105 immunolabeling of the ventricular wall (C) identifies filopodia-guided processes (arrows) of endocardial sprouting. Desmin-positive reactions were exclusively found (D) in the dorsal wall of the venous sinus (arrows) and in the atrioventricular ring (arrowheads) (VS: venous sinus; RA: right atrium; RV: right ventricle; avc: atrioventricular cushion). CD31-positive endocardial (arrows) and vascular (arrowhead) endothelia were identified. α-SMA intense labeling of the venous sinus (VS) myocardium (arrow) but not of subepicardium (arrowhead).

**Figure 2:**
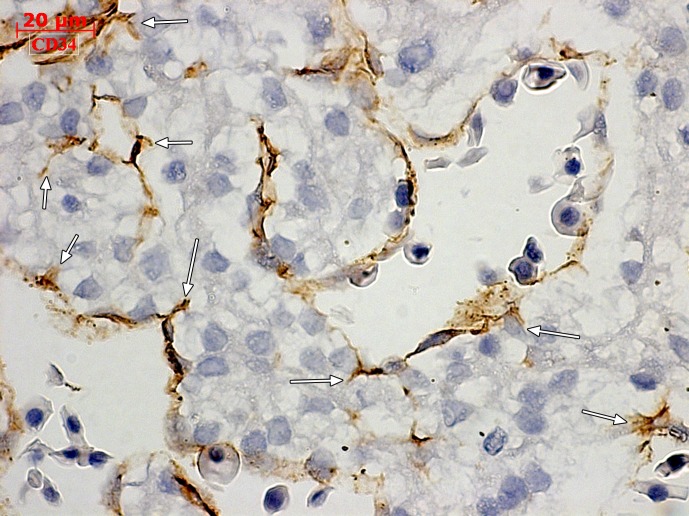
Human embryonic heart (43 days), CD 34 immune labeling. Immune labeling with CD34 antibodies of a 43 days human embryonic heart, oblique-sagittal cut at ventricle level. Endocardial tip cells are indicated (arrows).

In a 43 days embryo, the heart was identified, covered by the epicardial mesothelium layer, at the atrial and ventricular level. Both endocardial and epicardial cells were vimentin-positive. Moreover, vimentin labeled both subepicardial and intramyocardial interstitial cells. Atrial subepicardial endothelial tubes, distinctive from the epicardial mesothelium, but apparently connected to it, were vimentin-positive and better represented near to the atrioventricular canal from the inferior part of the heart. At the ventricular level, mostly on the ventral surface and distally, towards the *apex cordis*, we found large successive vascular canals covered by epicardial mesothelium; the walls of these canals that appeared to be blood islands, were strongly vimentin-positive and were apparently involved in processes of sprouting angiogenesis (**Fig. 1**). CD34 labelled the endocardium but not the epicardium, did not label any stromal cells, and only discretely labelled the walls of the ventricular subepicardial vascular canals—an immune-staining pattern similar to CD31 (**Fig. 1**). We also assessed intramyocardial processes of sprouting angiogenesis guided by ETCs, but they did not appear to be anatomically linked to vascular or endocardial endothelia. Intratrabecular sinusoids were coated by CD34+/CD105+ endocardial endothelial cells (**Fig. 1**), with filopodia projected in the myocardium beneath (**Fig. 2**). This morphology allowed us to consider them as *endocardial tip cells*. Desmin-positive mural labelling was assessed in the posterior wall of the venous sinus and the atrioventricular ring (**Fig. 1**). α-SMA antibody did not label subepicardium in this stage, nor the ventricle wall, but an intense labeling was found in the *sinus venosus* myocardial layer (**Fig. 1**).

**Figure 3:**
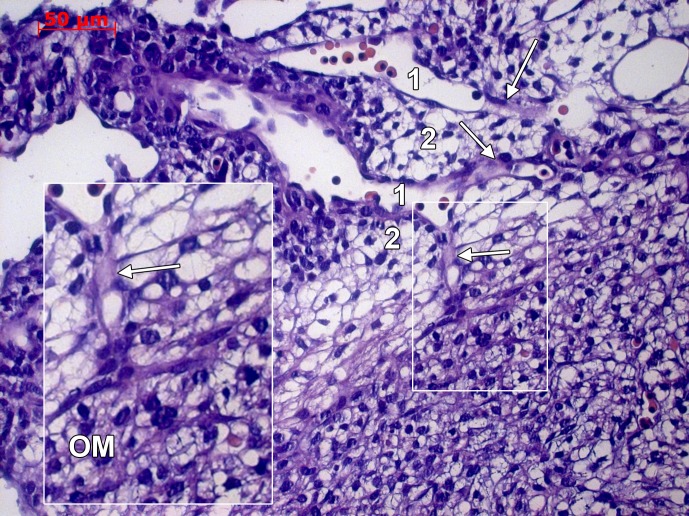
Human embryonic heart (48 days), hematoxylin-eosin staining. Hematoxylin-eosin stained heart in a 48 days embryo. The aortic sinus (1) and cushions (2) are indicated. Primitive coronary arteries emerge (arrows) the coronary sinus and dichotomize within the outer myocardium (OM, inset).

**Figure 4:**
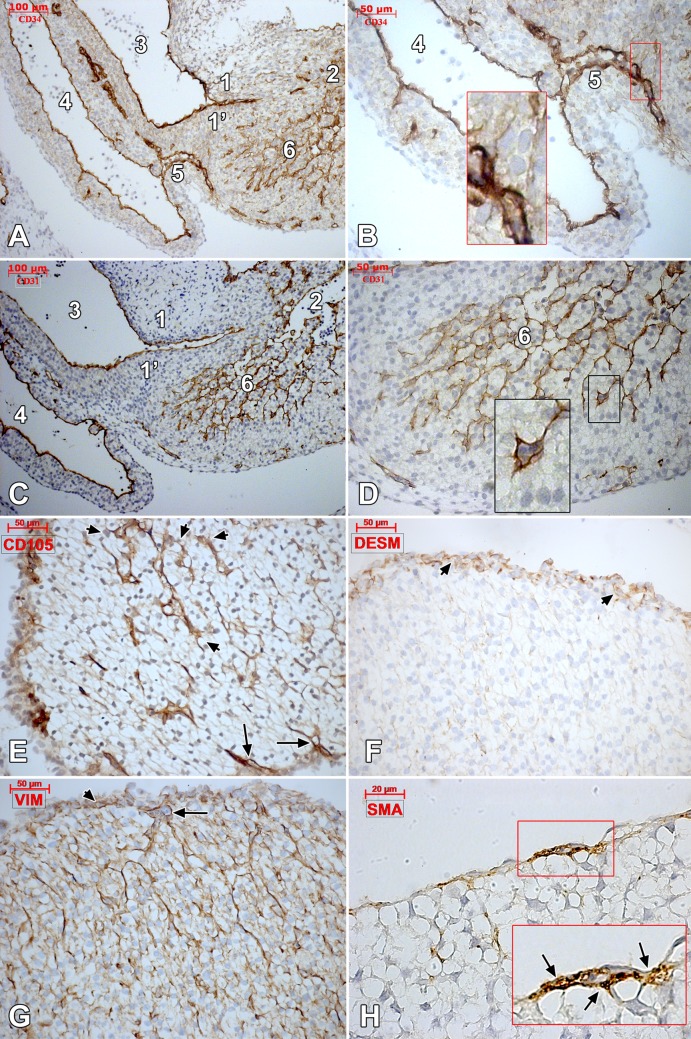
Human embryonic heart (48 days), CD 34 and CD 31 immune labeling. Oblique sagittal cut of a 48 days human embryonic heart. Immune labeling of successive slides with CD34 (A, B) and CD31 (C, D) antibodies (1, 1’.atrioventricular cushions; 2.ventricle; 3.primitive atrium; 4.*sinus venosus*; 5.coronary sinus; 6. endocardial-derived endothelial network in the ventricular wall); insets depict filopodia-projecting endocardial tip cells of the *sinus venosus* wall (B) and ventricular wall (D). CD105-positive epicardially-derived endothelial tubes (arrows) and intramyocardial endothelia (arrowheads) are indicated in (E). A continuous desmin-positive reaction is identified (F) in the epicardium (arrowheads), but not intramyocardially. Vimentin labeled (G) both the endocardial endothelia (arrow) and the epicardial stroma (arrowhead). Subepicardial vessels are coated by α-SMA-positive pericytes (H, inset, arrows).

**Figure 5:**
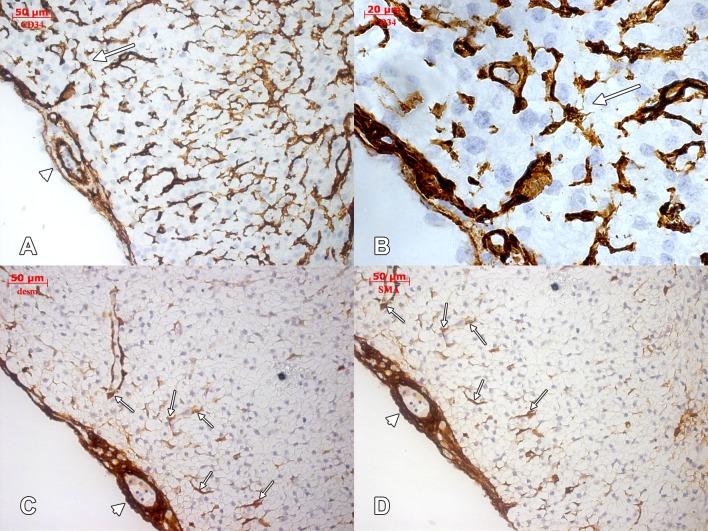
Human embryonic heart (56 days), CD 34, desmin and α-SMA immune labeling. Immune labeling for CD34 (A, detail in B), desmin (C) and α-SMA (D) of the future posterior interventricular groove in a 56 days human embryonic heart, oblique-sagittal cut. Endothelial sprouts guided by tip cells (arrow, A and B) invade the outer myocardium. The subepicardial vessels are embedded in a myoid stroma, α-SMA- and desmin-positive (arrowhead in C and D). Myoid cells (arrows in C and D) contact the endothelial sprouts within the outer myocardium.

In late stage embryos (6–8 weeks), the aortic sinuses beneath the aortic cushions were found sending off a number of arteries that penetrated the subepicardium and the outer myocardium further; where they were dichotomizing (**Fig. 3**). The coronary sinus was identified leaving the *sinus venosus* and branching into the *crista terminalis* and the inferior surface of the ventricle (**Fig. 4**). Endocardial and vascular endothelia were CD31+/CD34+/CD105+/vimentin+ (**Fig. 4**) and featured filopodia-projecting endothelial cells. Apparently, a rich endothelial network was supplied by the endocardial endothelium within the inner myocardium (**Fig. 4**). The outer part of the myocardium was supplied by stromal-embedded subepicardial vessels (**Figs. 4 and 5**), that were sending myocardial branches involved in active processes of sprouting, as proved by ETCs. Within the subepicardium α-SMA+/desmin+ myoid coats of the endothelial tubes were found (Figs. 4 **and** 5). In the outer part of the myocardium, α-SMA+/desmin+ cells were apposed to the endothelial sprouts (**Fig. 5**).

**Figure 6:**
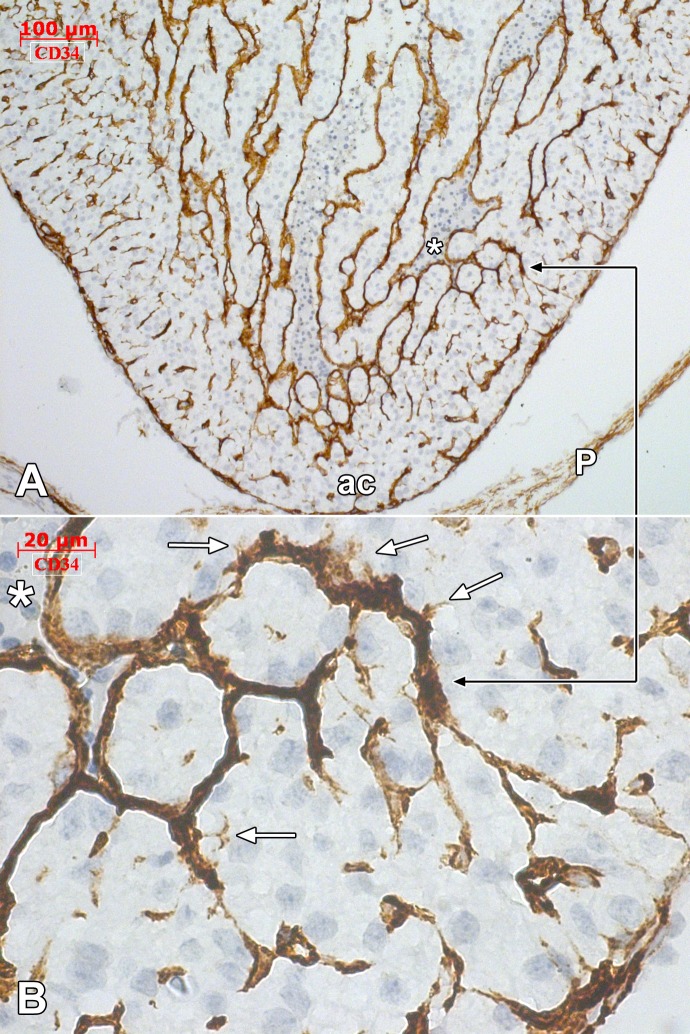
Human embryonic heart (56 days), CD 34 immune labeling. Immune labeling with CD34 antibodies of a 56 days human embryonic heart, oblique-sagittal cut. General view (A) with detailed area in (B), indicated by the black connector. The CD34 positive endocardial cells cover the ventricular cavity (*). Endocardial tip cells (white arrows) are identified projecting filopodia within the myocardium. The endocardially-derived endothelial networks advance towards the epicardially-derived endothelial networks. ac: *apex cordis*; P:pericardium.

**Figure 7:**
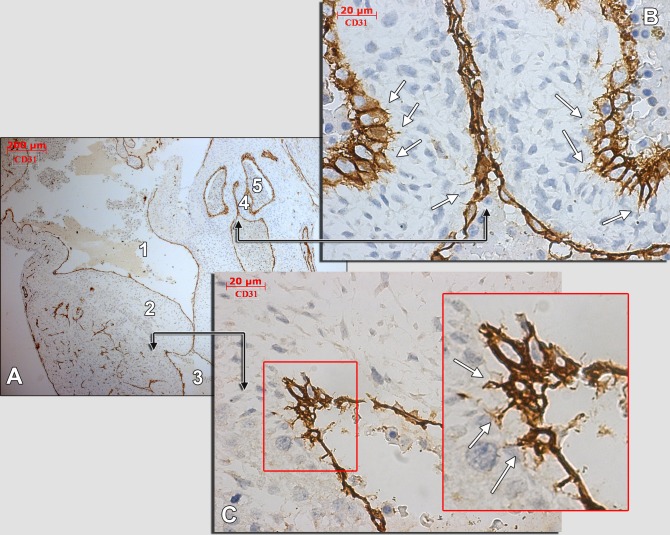
Human embryonic heart (56 days), CD 31 immune labeling. Immune labeling with CD31 antibodies of a 56 days human embryonic heart, oblique-sagittal cut. A general view is presented in (A) and detailed in (B) and (C) (corresponding fields are indicated by black connectors). An area in (B) is magnified (inset). The white arrows indicate endocardial tip cells projecting moniliform filopodia within the subendocardial stroma. 1.atrioventricular canal; 2.atrioventricular cushion; 3.ventricle; 4.aortic cushion; 5.aortic sinus.

Within the ventricular walls the general pattern appeared as follows (**Fig. 6**): the outer myocardium was supplied by subepicardial vessels and the endothelial sprouts were guided by ETCs, while the inner myocardium was supplied by endocardial-derived endothelial networks, the endothelial sprouts being guided by endocardial tip cells. A different pattern was encountered in the atrioventricular and arterial cushions: these were seemingly completely devoid of microvessels but endocardial tip cells were consistently projecting filopodia in the subendocardial tissues (**Fig. 7**).

**Figure 8:**
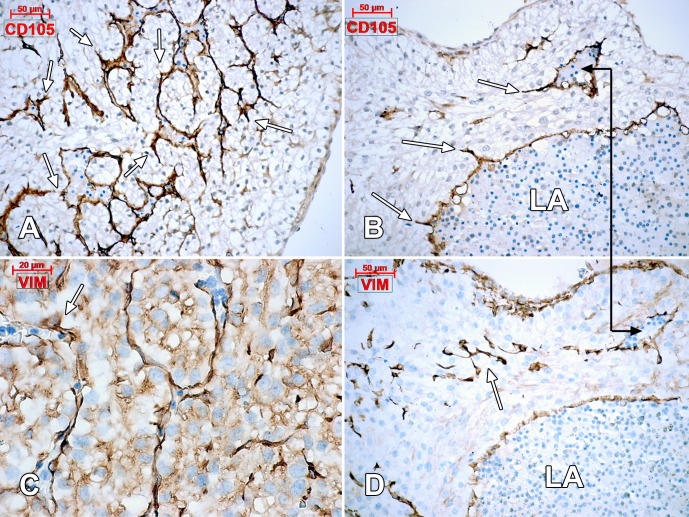
Human embryonic heart (56 days), CD105 (A, B) and vimentin (C, D) immune labeling. Intramyocardial endothelial networks result from processes of sprouting angiogenesis (arrows) of the underlying CD105-positive and vimentin-positive endocardium. The connector indicates corresponding areas of the left atrium (LA) wall.

Although the atrial subepicardial layer was not distinctive, we found scarce endothelial tubes beneath the epicardial mesothelium, but they were apparently not involved in any intramyocardial branching or sprouting. Instead, the endothelial networks of the atria walls were supplied by atrial endocardium and endothelial sprouts guided by endocardial tip cells (**Fig. 8**).

## Discussion

Phng proved that ETCs are generating lamellipodia that are able, when ETCs filopodia formation is inhibited, to drive the ECs migration [12]. The lack of studies identifying ETCs filopodia in light microscopy should not exclude an active process of sprouting angiogenesis; *per contra,* finding ETCs filopodia is a strong argument for an ongoing angiogenesis.

There are studies proving CD34 and/or CD31 positive phenotypes for ETCs [4, 5, 20]. Moreover, CD105 (endoglin) is up-regulated in activated endothelial cells thus being an appropiate marker for identifying endothelial sprouts [21] and ETCs. Thus, there are no doubts about the positive labeling for these markers of both vascular, and endocardial ETCs in our study. Vimentin also appeared as reliable marker for ETCs. Although the prolongations of ETCs could be morphologically misdiagnosed as processes of telocytes [11], a peculiar cell phenotype, but the location of the cell bodies answers this issue. In this study, we did not found stromal cells positive for CD34 to raise any suspicion of a stem/progenitor cell phenotype as it was discussed elsewhere [22]. It is however difficult to distinguish cells undergoing sprouting angiogenesis of endocardial cells of trabeculae; further studies should attempt to scan consecutive sections and then 3D render individual cells. Thus, invading cells would be distinguishable from surrounding squamous endocardial cells. One way that sprouting cells can be distinguished from endocardial cells is that they exhibit filopodia.

The origin of the pre-endocardial cells is still not fully clarified [23]. While a mesodermal origin is undisputed, the exact outset either from a heart field or from a neighbor area is yet to be determined [23]. Several studies established various mesodermal territories as origin for progenitors of different cardiac tissues; these heart fields are well-characterized, at least in what concerns their derivatives [24]. It was also suggested a common myocardial-endocardial progenitor [25]. Recent studies established however that the second heart field contains distinctive progenitors, myocardial and endocardial; moreover, convincing evidence was brought for an endocardial origin, at least partly, from vascular endothelial cells [26]. In this regard, we are tempted to speculate that at least some of the endocardial endothelial cells seem pre-qualified to participate in processes of sprouting angiogenesis during development, and to generate vascular endothelial cells, as it was previously showed [16].

One of the contributors of the cardiac microvascular bed is the endothelial lining of the *sinus venosus* [27]. This is undoubtfully supported by our study. Moreover, the coronary sinus was found branching within the *crista terminalis* and subepicardially, on the inferior wall of the right ventricle. Anatomically, the *sinus venosus* endothelium could be regarded as the primary source for subepicardial venous endothelia, which it further develops by sprouting angiogenesis. This is in accordance with Red-Horse et al. (2010), who found in mice embryos a vascular plexus—that originated from the *sinus venosus* and expanded by sprouting angiogenesis—to invade the underlying myocardium (similarly, a ventral plexus developed). [28]. They also took into account early studies supporting that the coronary arteries bud from the aorta in humans and other mammals but rejected those theories by referring to more recent chick-quail chimeras studies [28]. We gathered however direct proofs of aortic-linked primordia of coronary arteries in human embryos, as well as of their subepicardial course and branching within the outer myocardium, but estimation of their general distribution is difficult. The theory of the origin of the coronary endothelial cells from proepicardium/subepicardium, supported by other authors [28–34] could not be rejected. This is because, on one hand, interspecies differences could not be ignored and, on other hand, a heterogeneity of the precursor cell population, as proven for the outflow tract [35], could, and should not be completely excluded. In human embryos lineage tracing is not feasible thus the results of this study could not be used to discriminate between theories.

The contribution of the epicardially-derived stromal cells to the vascular smooth muscle cells and pericytes is supported by previous studies [36–40]. However, although desmin and α-SMA are known markers for pericytes [4, 41, 42], their heterogeneous phenotype make the unambiguous identification of pericytes a challenge [40]. Vascular and perivascular smooth muscle cells are also recruited from the intramyocardial epicardially-derived stroma. In this regard, we could not identify specific studies of coronary vascular development to support this hypothesis, but previous evidence suggested that signals originated from myocardium could control the regionally restricted process of coronary vasculogenesis [43]. It should be considered that, although there is a consensus regarding the mesenchymal origin of pericytes, these cells can also result by transdifferentiation of endothelial cells [18, 40]. It was proved that, during sprouting angiogenesis, fibroblasts settle down on sprouts and are converted to pericytes as they are embedded within the endothelial basal lamina, thus reinforcing the endothelial walls of the sprouts [44]. Basically, ETCs recruit pericytes [45] that is an organ-specific process during development [46]. VEGF accelerates pericyte coverage of endothelia [46] but, on the other hand, the endocardially derived-VEGF receptors inhibit coronary angiogenesis [16, 18] and a subendocardial stroma is not available as a pool of smooth muscle cells; these opposed mechanisms could favor, hypothetically, a final prevalence of the subepicardial-derived vessels within myocardium, as compared to the endocardially-derived ones. It is therefore reasonable to speculate a role of the epicardially-derived fibroblasts, subepicardial and intramyocardial, in stabilizing the endothelial sprouts. If these may be consistent with the ventricular microcirculatory bed, a different morphological pattern seems related to the atrial microcirculatory bed, perhaps determined by the fact that the atrial epicardium does not show an epithelial-mesenchymal transformation, which governs the formation of the subepicardium [37]. With a poorly represented atrial subepicardium, with scarce submesothelial vessels, the endothelial networks of the atrial walls were consistently derived from endocardium. Therefore, while the atrial microcirculatory bed seems to be derived mostly from the endocardium, the ventricular one is derived from both subepicardium and endocardium. This completes previous evidence of the contribution of the ventricular endocardial cells to the cardiac microvasculature [16, 18].

The *in situ* evidence we gathered by this study leads to a series of anatomical hypotheses: (1) primordia of the venous and arterial main vascular trunks derive from both *sinus venosus* and aorta, respectively, and penetrate the subepicardium; (2) subepicardial endothelial tubes form and elongate the main vascular trunks of the heart, most likely except for the atria; (3) the microvascular network of the outer ventricular myocardium derives from the subendotelial vessels and further develops by ETCs—guided angiogenesis; (4) within the inner ventricular myocardium and the atrial myocardium angiogenic sprouts are guided by endocardial tip cells; (5) microvascular networks of the inner and outer myocardium are supposed to further connect; however, the distinction between arterial and venous beds is difficult. As it was previously shown, we are still far from understanding how vascular pattern and differentiated identity are established [47].

It was shown in zebrafish embryos that, in a temporal sequence, from aorta derives a primary network of vascular segments; then vascular sprouts, vein-derived, interconnect with the primary network to initiate vascular flow [48]. A similar mechanism may be presumed for the heart; the arterial end of the cardiac microcirculations is undoubtfully linked to the aorta, *via* the primordia of the coronary arteries, the venous end emerges from the venous sinus, the subepicardial endothelia adds length to the subepicardial vessels, while endocardial-derived endothelial networks could link to both arterial and venous tubes to establish a complete cardiac circulation.

## Supporting Information

S1 Fig Negative controls (primary antibodies omitted). EDTA (A) and citrate (B) techniques.
(TIF)
