# Correction: Endocardial Tip Cells in the Human Embryo - Facts and Hypotheses

**DOI:** 10.1371/journal.pone.0121036

**Published:** 2015-03-27

**Authors:** The PLOS ONE Staff

There is an error in affiliation 5 in the PDF version of the published article for author Mihnea I. Nicolescu. Affiliation 5 should be: Laboratory of Molecular Medicine, “Victor Babeş” National Institute of Pathology, Bucharest, Romania.

Reference 22 has been published. The full reference is: Diaz-Flores L, Gutierrez R, Garcia MP, Saez FJ, Diaz-Flores L Jr, et al. (2014) CD34+ stromal cells/fibroblasts/fibrocytes/telocytes as a tissue reserve and a principal source of mesenchymal cells. Location, morphology, function and role in pathology. Histol Histopathol 29: 831–870. pmid:24488810

Fig. 1 is incorrect. Please view the correct Fig. 1 here.

**Fig 1:**
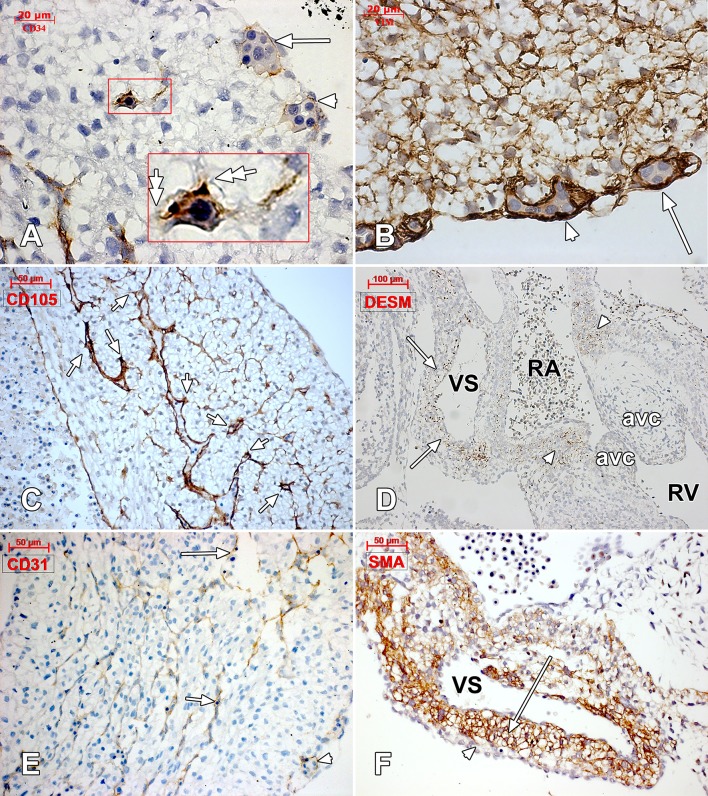
Human embryonic heart (43 days), CD 34, vimentin, CD105, desmin and CD31 immune labeling. Immune labeling with CD34 (A) and vimentin (B) antibodies of a 43 days human embryonic heart, oblique-sagittal cut at ventricle level. Corresponding epicardial vascular canals are indicated (white arrrows and white arrowheads). The walls of these canals seem to acquire a CD34-positive phenotype and are vimentin-positive. In (A) an active intramyocardial process of sprouting angiogenesis is detailed (inset), being guided by tip cells (double-headed arrows). CD105 immunolabeling of the ventricular wall (C) identifies filopodia-guided processes (arrows) of endocardial sprouting. Desmin-positive reactions were exclusively found (D) in the dorsal wall of the venous sinus (arrows) and in the atrioventricular ring (arrowheads) (VS: venous sinus; RA: right atrium; RV: right ventricle; avc: atrioventricular cushion). CD31-positive endocardial (arrows) and vascular (arrowhead) endothelia were identified. α-SMA intense labeling of the venous sinus (VS) myocardium (arrow) but not of subepicardium (arrowhead).
