# Supplementary figures and images for: Endocardial Tip Cells in the Human Embryo – Facts and Hypotheses
Source: PLoS One. 2015 Jan 24;10(1):e0115853. doi: 10.1371/journal.pone.0115853 (PMC4305311; doi:10.1371/journal.pone.0115853)

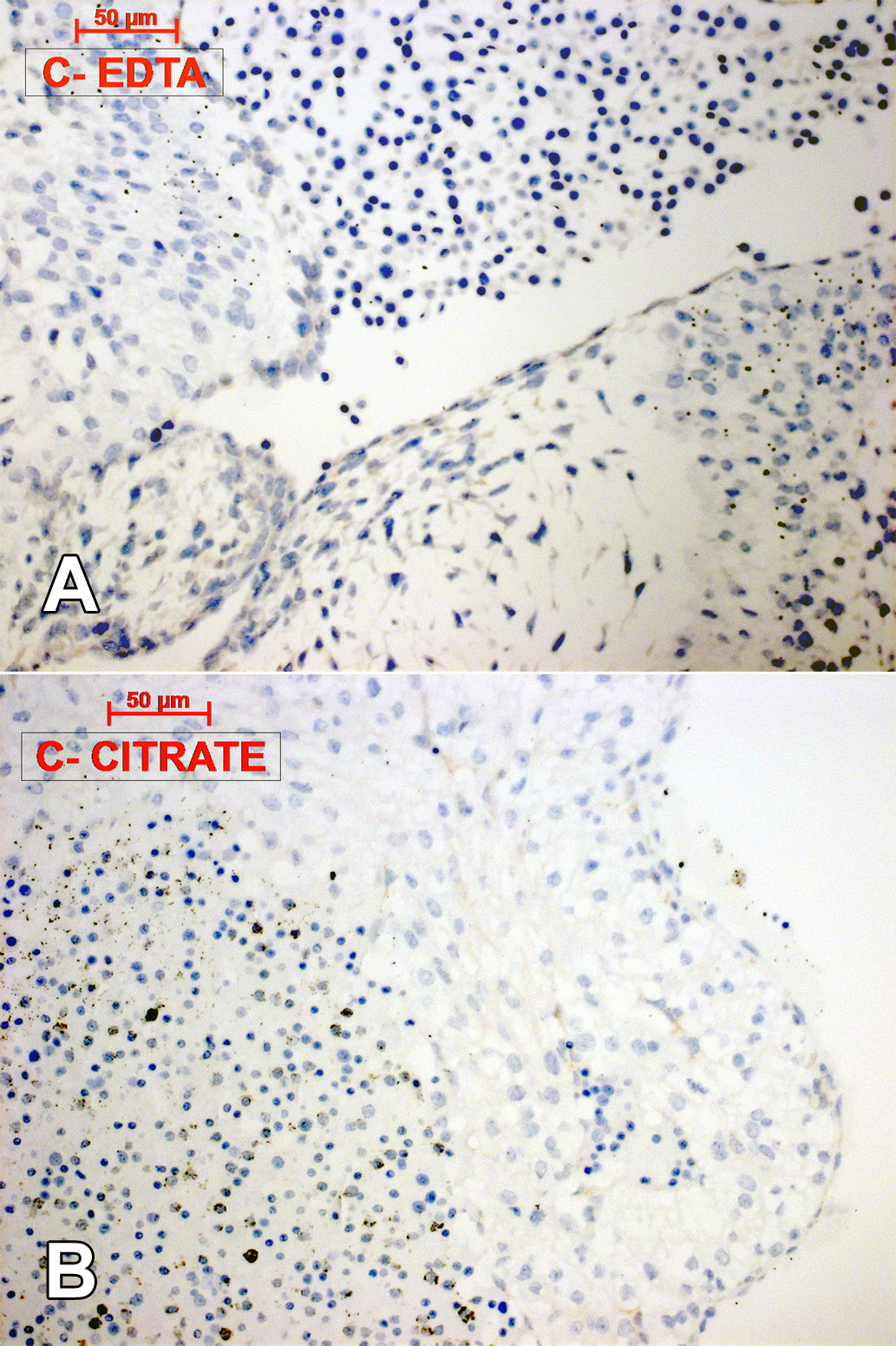

Supplement: S1 Fig — EDTA (A) and citrate (B) techniques. (TIF) [file pone.0115853.s001.tif]
